# Systemic Myostatin Inhibition via Liver-Targeted Gene Transfer in Normal and Dystrophic Mice

**DOI:** 10.1371/journal.pone.0009176

**Published:** 2010-02-11

**Authors:** Kevin J. Morine, Lawrence T. Bish, Klara Pendrak, Meg M. Sleeper, Elisabeth R. Barton, H. Lee Sweeney

**Affiliations:** 1 Department of Physiology, University of Pennsylvania School of Medicine, Philadelphia, Pennsylvania, United States of America; 2 Section of Cardiology, University of Pennsylvania School of Veterinary Medicine, Philadelphia, Pennsylvania, United States of America; 3 Department of Anatomy and Cell Biology, University of Pennsylvania School of Dental Medicine, Philadelphia, Pennsylvania, United States of America; University of Florida, United States of America

## Abstract

**Background:**

Myostatin inhibition is a promising therapeutic strategy to maintain muscle mass in a variety of disorders, including the muscular dystrophies, cachexia, and sarcopenia. Previously described approaches to blocking myostatin signaling include injection delivery of inhibitory propeptide domain or neutralizing antibodies.

**Methodology/Principal Findings:**

Here we describe a unique method of myostatin inhibition utilizing recombinant adeno-associated virus to overexpress a secretable dominant negative myostatin exclusively in the liver of mice. Systemic myostatin inhibition led to increased skeletal muscle mass and strength in control C57 Bl/6 mice and in the dystrophin-deficient *mdx* model of Duchenne muscular dystrophy. The *mdx* soleus, a mouse muscle more representative of human fiber type composition, demonstrated the most profound improvement in force production and a shift toward faster myosin-heavy chain isoforms. Unexpectedly, the 11-month-old *mdx* diaphragm was not rescued by long-term myostatin inhibition. Further, *mdx* mice treated for 11 months exhibited cardiac hypertrophy and impaired function in an inhibitor dose–dependent manner.

**Conclusions/Significance:**

Liver-targeted gene transfer of a myostatin inhibitor is a valuable tool for preclinical investigation of myostatin blockade and provides novel insights into the long-term effects and shortcomings of myostatin inhibition on striated muscle.

## Introduction

Myostatin or Growth Differentiation Factor 8 (GDF-8) is a member of the pleiotrophic transforming growth factor β family and is a potent negative regulator of skeletal muscle mass. Myostatin is highly conserved across species and when functionally inactivated results in profound increases in skeletal muscle mass in cows, dogs, sheep, mice and humans [1]. Conversely, ectopic overexpression of myostatin induces skeletal muscle atrophy [2]. A multitude of experiments have demonstrated that the absence of myostatin due to genetic deletion or inhibition results in increased muscle mass [3]–[6]. While the effect of myostatin inhibition on skeletal muscle mass and morphology is dependent on the route, timing and mechanism of blockade, invariably there is an increase in muscle size due to the growth of muscle fibers (hypertrophy) and/or an increased number of muscle fibers (hyperplasia). When myostatin is genetically deleted or inhibited by transgenic expression of the myostatin binding protein follistatin, there is a more profound increase in muscle mass and both hypertrophy and hyperplasia of fibers is observed [4], [5]. Postnatally, myostatin inhibition achieved by neutralizing antibodies directed against myostatin administered in normal C57 Bl/6 mice and in the *mdx* mouse model of Duchenne muscular dystrophy leads to muscle growth due to hypertrophy and not hyperplasia [3], [6].

A common feature of murine dystrophic animal models is the compensatory regeneration of muscle fibers due to satellite cell activation, proliferation and fusion to existing muscle fibers or *de novo* formation of myofibers. Hence myostatin based therapies hold great promise for the muscular dystrophies due to their ability to increase fiber size, enhance regeneration and regulate muscle fibrosis [3], [7], [8]. Myostatin inhibition has resulted in significant amelioration of dystrophic pathology in a mild dystrophic model (*mdx*), mixed findings in a severe dystrophic model (*dsg)* or no improvement in severe dystrophic models (*gsg, dy*) [5], [9]–[11]. That myostatin inhibition does not improve severe muscular dystrophy is not surprising as myostatin cannot correct the underlying genetic defect. For the muscular dystrophies, myostatin inhibition could be utilized to augment correction of the primary defect by promoting short term gains of muscle mass. Increased muscle mass may protect against contraction induced damage by reducing the amount of muscle recruited to produce a given amount of force [12]. Myostatin modulation also has therapeutic potential for disease states that involve the loss of normal muscle mass, such as cancer cachexia, disuse atrophy, sarcopenia and microgravity exposure. However, there is the possibility that myostatin inhibition in the heart could interfere with cardiac adaptation to underlying cardiac disease that may occur with aging and a subset of the muscular dystrophies.

Previous preclinical studies in the *mdx* mouse model of Duchenne muscular dystrophy targeting the myostatin pathway have utilized neutralizing antibodies, myostatin propeptide injection and recombinant adeno-associated virus (AAV) mediated expression of a myostatin inhibitor from multiple tissues [5], [13], [14]. These studies all showed skeletal muscle improvement although the animals were only followed for 3–4 months following initiation of treatment. As the muscular dystrophies result in progressive pathology of skeletal muscle, long term studies are crucial to determine if proposed treatments will correct the pathology over time and to assess the effect of myostatin inhibition on cardiac function. At nine months of age *mdx* mice first develop signs of a progressive dilated cardiomyopathy, an age that was not analyzed in these studies [5], [13], [14]. Qiao et al. recently reported that the AAV mediated expression of a mutated myostatin propeptide increases muscle size and improves pathology in *mdx* animals [14]. While they suggested that this was due to liver secretion, immunoblotting did not show expression of the propeptide in the liver and RT-PCR showed expression of the transgene in the heart as well as the liver. As a ubiquitous promoter was used in this study, it is possible that propeptide is not being expressed in the liver but from the heart as well as other unscreened skeletal muscles. Haidet et al. used intramuscular injections of AAV to overexpress endogenous proteins that inhibit myostatin [15]. A limitation of this study is that none of the inhibitors was specific for myostatin. For example, follistatin and follistatin like related gene (FLRG) bind to activin in addition to myostatin and GDF associated serum protein 1 (GASP1) also contains protease domains [16]–[19]. The observed beneficial effects on muscle growth may depend on signaling pathways other than myostatin that are not specific to muscle, and the modulation of non-muscle tissue physiology may limit clinical application.

While there has been considerable interest in utilizing myostatin inhibition to ameliorate muscular dystrophy and other disorders, comprehensive long term studies of postnatal myostatin inhibition have not yet been reported. An evaluation including functional assessment of slow and fast muscle types as well as the diaphragm and heart is lacking. The diaphragm is the most severely dystrophic skeletal muscle in the *mdx* mouse and is the mouse muscle most representative of human disease progression. Direct injection of all of these tissues is not possible and dystrophic muscle does not support long term AAV transduction without membrane stabilization. The loss of AAV transduction over time is most likely due to muscle fiber turnover as evidenced by the loss of AAV mediated IGF expression after four months in *mdx* muscle (unpublished observations by E.R. Barton and H.L. Sweeney) and a similar loss of AAV mediated myostatin propeptide expression after six weeks in the highly regenerative alpha-sarcoglycan null model [20], [21].

To address these questions we developed a method that achieves persistent myostatin inhibition through a single dose of AAV to overexpress a novel myostatin inhibitor from the liver of C57 Bl/6 and *mdx* mice. C57 Bl/6 mice were treated as neonates or young adults then examined three months post injection to determine the effect of myostatin inhibition on muscle mass in normal animals. *Mdx* mice were injected as neonates or one month of age and examined at four and eleven months of age to assess the effect of myostatin inhibition at early and later stages of muscle pathology. A functional and histological assessment of the soleus, extensor digitorum longus (EDL), diaphragm and heart were made to determine the effect of myostatin inhibition systemically. These findings have important implications for future therapies that utilize the myostatin pathway and for the regulatory role of myostatin in skeletal and cardiac muscle.

## Results

**Figure 1:**
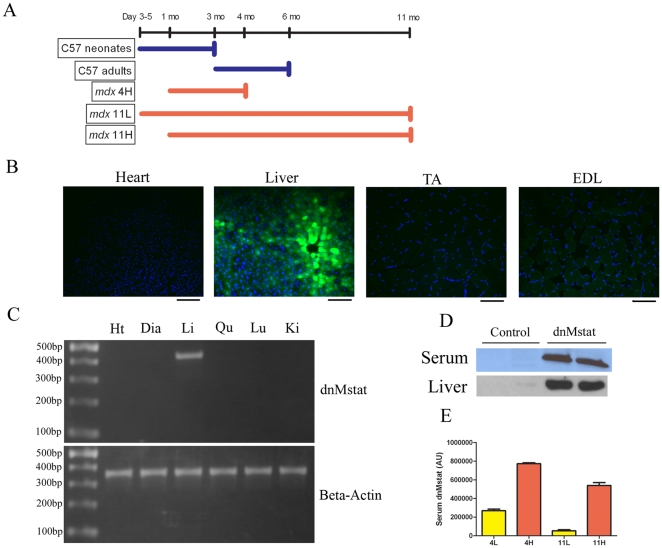
Systemic viral delivery and dnMstat expression. The viral construct used in all experiments consists of a liver specific promoter (LSP) paired with dominant negative myostatin (dnMstat). A similar construct was prepared by substituting GFP for dnMstat. (a) Experimental design for C57 Bl/6 and *mdx* groups injected intravenously with AAV 2/8 LSP.dnMstat. The C57 neonates group was injected at day 3–5 of life and sacrificed at three months of age while the C57 adults group was injected at three months of age and sacrificed at six months of age. The *mdx* 4H group was injected at one month of age and sacrificed at four months of age. The *mdx* 11L and 11H groups were sacrificed at eleven months of age and injected as neonates (11L) or at one month of age (11H). AAV 2/8 LSP.GFP or AAV 2/8 LSP.dnMstat was injected into the tailvein of adult C57 Bl/6 mice to assess the specificity of the promoter and to confirm overexpression of dnMstat. (b) Following injection of AAV 2/8 LSP.GFP, GFP fluorescence was only observed in the liver. (c) Tissue specificity of transgene expression was further assessed by RT-PCR following injection of AAV 2/8 LSP.dnMstat. RNA was isolated from treated heart (Ht), liver (Li), diaphragm (Dia), quadriceps (Qu), lung (Lu) and kidney (Ki), treated with DNase to remove residual AAV vector genomes and reverse transcribed. PCR was performed on the resulting cDNA to assess the expression of dnMstat (∼450 bp) and beta-actin as a control (∼350 bp). Transgene expression was detected solely in the liver. (d) dnMstat was detected in the serum and liver by immunoblotting with an antibody directed against the myostatin N-terminus. (e) Serum levels of dnMstat were measured at the experimental endpoints. Circulating levels of the inhibitor were approximately three times higher in the *mdx* 4H group than the *mdx* 4L group. The absolute difference in expression level was maintained at eleven months of age in the 11L and 11H groups. Note that the *mdx* 4L group was not analyzed further Scale bar: 100 µm.

To express persistently a circulating myostatin inhibitor, a dominant negative myostatin propeptide was engineered (dnMstat), paired with a liver specific promoter (α_1_-antitrypsin promoter with ApoE enhancer, abbreviated LSP) and AAV psuedotype 2/8 LSP.dnMstat was produced. AAV pseudotype 2/8 has been previously shown to have excellent tropism for the liver and following intravenous injection demonstrates superior transduction of the liver in comparison to other AAV serotypes [22], [23]. The experimental design of the mouse studies is shown in Figure 1A. C57 Bl/6 mice were treated as neonates (n = 6 control, n = 6 treated, denoted C57 neonates) or as adults at three months of age (n = 6 control, n = 6 treated, denoted C57 adults) and analyzed three months following viral injection. The *mdx* mouse model of Duchenne muscular dystrophy was utilized to examine the effect of myostatin inhibition on the progression of muscular dystrophy. Levels of dnMstat were assessed in the serum of all *mdx* groups at sacrifice (**Fig. 1E**). At four months of age, there was a ∼3 fold increase in circulating inhibitor when mice were treated at one month of age compared to treatment as neonates. The difference in circulating inhibitor was maintained throughout the study, although both injection methods exhibited similar absolute decreases by eleven months of age. Viral injection in neonatal animals effectively resulted in a low dose of dnMstat while viral injection in one month old animals resulted in a high dose of dnMstat. For the purpose of simplifying discussion, we named the experimental groups to reflect the age of the animal at the experimental endpoint and dose of inhibitor. *Mdx* mice treated at one month of age and analyzed at four months of age (n = 6 control, denoted *mdx* 4C and n = 6 treated, denoted *mdx* 4H). A small group of *mdx* mice were injected as neonates and sacrificed at four months of age to determine the effect of age on viral expression and were not evaluated further (n = 4 treated, denoted *mdx* 4L). For the eleven month endpoint, mice were treated as neonates (n = 6, denoted *mdx* 11L) or one month of age (n = 4, denoted *mdx* 11H) with a common set of controls (n = 6, denoted *mdx* 11C).

**Figure 2:**
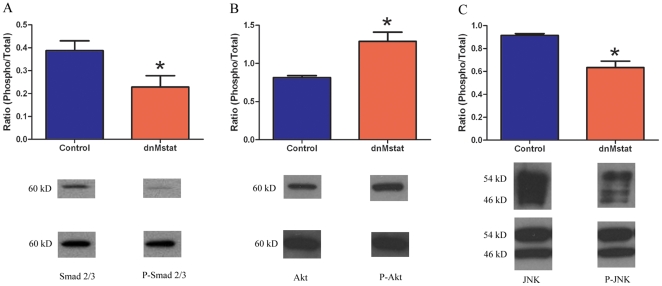
Signaling changes following short term myostatin inhibition in C57 Bl/6 mice. C57 mice were injected intravenously with AAV 2/8 LSP.dnMstat and after one week the animals were sacrificed. Immunoblotting for total and phosphorylated Smad2/3, Akt and JNK was performed on protein homogenates of quadriceps muscle using beta-actin as a loading control. In dnMstat treated C57 quadriceps there was a (a) 41% decrease in phosphorylated Smad 2/3, (b) 59% increase in phosphorylated Akt and (c) 31% decrease in phosphorylated JNK. Data represent mean±SD. * Statistically significant compared to control, p<0.05.

The specificity of the LSP was confirmed by intravenous delivery of 1E12 genome copies of AAV2/8 LSP.GFP. Expression of GFP was detected exclusively in the liver while no fluorescence was observed in the heart or skeletal muscle (**Fig. 1B**). The tissue distribution of transgene expression was further assessed by reverse transcriptase polymerase chain reaction in six different tissues. Expression of the transgene was only detected in the liver (**Fig. 1C**). AAV2/8 LSP.dnMstat was injected into the tailvein of adult C57 Bl/6 mice and high levels of the inhibitor were detected in the blood as well as the liver after one week (**Fig. 1D**). C57 Bl/6 animals were sacrificed one week after viral injection to assess acute changes in the activity of key intracellular regulators of muscle growth. In treated quadriceps there was a decrease in phosphorylated Smad 2/3, a decrease in phosphorylated JNK and an increase in phosphorylated Akt (n = 4) (**Fig. 2**). These results demonstrate that the liver was a viable target for AAV mediated overexpression of a circulating myostatin inhibitor.

**Figure 3:**
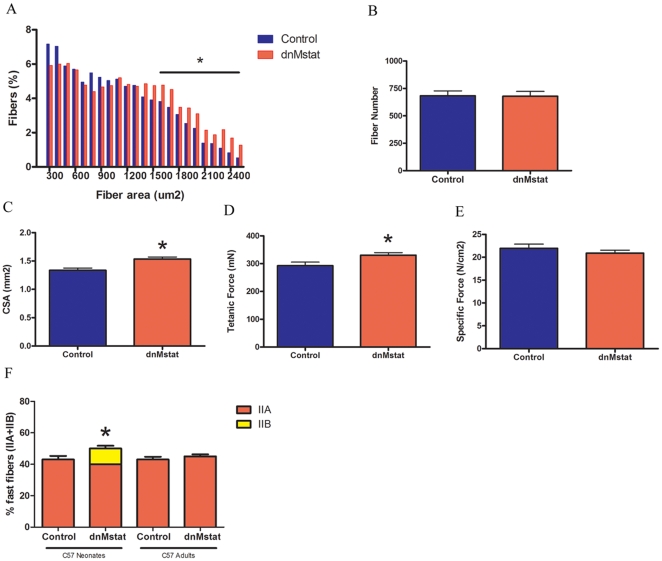
Effect of systemic myostatin inhibition in C57 Bl/6 mice. Adult female C57 Bl/6 mice were injected intravenously with AAV2/8 LSP.dnMstat and analyzed three months after injection at six months of age (C57 adults). A second group of animals was injected via a subxyphoid approach at postnatal day 3–5 with the same virus and analyzed at 3 months of age (C57 neonates). The fiber area distribution, fiber number and functional properties of the EDL were analyzed in the C57 adults group. (a) In the dnMstat group there was a rightward shift in the fiber size distribution indicative of an increase in fiber area. (b) The number of fibers in the midbelly cross section of the EDL was unchanged. (c-e) There was an increase in cross sectional area (CSA) of the EDL with a proportionate increase in tetanic force resulting in no change in specific force. (f) The fiber type distribution of the soleus was unchanged in the C57 adults group while significantly more type IIB fibers were found in the C57 neonates group. Data represent mean±SD. * Statistically significant compared to control, p<0.05.

The efficacy of myostatin inhibition by dnMstat was assessed by analysis of muscle size and function of C57 Bl/6 animals overexpressing dnMstat. Expression of dnMstat resulted in a 14–22% increase of muscle mass of the quadriceps, tibialis anterior and extensor digitorum longus (EDL) muscles in the C57 adults group while a 32–43% gain in these muscles was observed in the C57 neonates group (**Table 1**). Thus a more pronounced increase in muscle mass in non-dystrophic animals was observed when inhibition was initiated early in the lifespan. The wet weight of the soleus and heart did not change in both C57 Bl/6 groups. In the C57 adults group, the EDL underwent hypertrophy without hyperplasia as indicated by a rightward shift in the fiber area distribution without a change in fiber number (**Figs. 3A** **, b**). The EDL exhibited an approximately 15% increase in cross sectional area (CSA) and tetanic force (**Fig. 3C, D**). No change in force production normalized to CSA (specific force) was observed (**Fig. 3E**). Taken together, these data indicate expression of dnMstat resulted in increased muscle mass, strength and hypertrophy without hyperplasia of normal skeletal muscle.

**Table 1:** Effect of systemic myostatin inhibition on muscle mass in C57 Bl/6 mice.

	Control Adults	dnMstat Adults	Control Neonates	dnMstat Neonates
Body weight (g)	22.7±0.7^a^	24.5±2.2	221.1±1.5	23.2±1.8
Quadriceps (mg)	158.1±15.6	192.9±7.9^b^	145.5±9.1	208.1±37.6^b^
Tibialis anterior (mg)	40.3±3.5	45.9±3.1^b^	40.2±1.9	53.9±1.21^b^
EDL (mg)	8.1±0.6	9.4±0.7^b^	8.9±0.4	11.8±0.4^b^
Soleus (mg)	8.2±0.4	8.8±0.9	7.4±0.2	8.7±0.2
Heart (mg)	102.2±3.2	107.3±5.1	104.9±4.2	108.6±4.9

a Data represent mean ± SD for muscles from sex and age matched control (n = 6) and treated mice (n = 6).

b Statistically significant compared to control, p<0.05.

To clarify the effect of myostatin inhibition in the soleus, fiber type distribution was evaluated in the C57 Bl/6 groups. There was no effect of dnMstat on soleus fiber type in the C57 adults group (**Fig. 3F**). However there was a shift toward faster fiber types in the C57 neonates group as 10% of the treated muscle fibers were MHC type IIB whereas no type IIB fibers were detected in control muscle.

**Figure 4:**
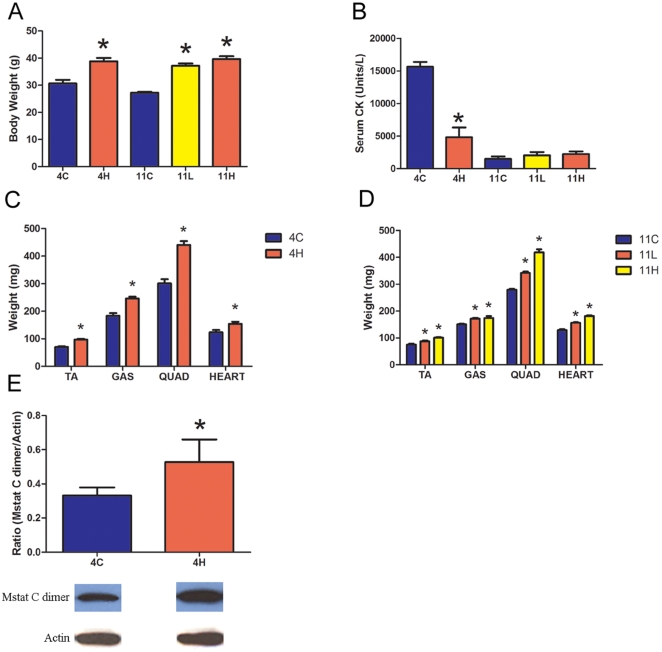
Effect of myostatin inhibition on body weight, serum creatine kinase and muscle weight in *mdx* mice. (a) Body weight was increased in all dnMstat treated groups. (b) Serum creatine kinase (CK) was reduced in the *mdx* 4H group in comparison to four month old *mdx* controls (4C), however it was not corrected in the *mdx* 11L and *mdx* 11H groups in comparison to eleven month old *mdx* controls (11C). (c) In the *mdx* 4H group, dnMstat overexpression increased the wet weight of the tibialis anterior (TA), gastrocnemius (GAS) quadriceps (QUAD) and heart. (d) At eleven months of age in the *mdx* 11L and 11H groups, these muscles continued to demonstrate increased mass. (e) Immunoblotting of *mdx* 4H quadriceps for the myostatin C terminus and beta-actin as a loading control revealed increased mature myostatin C dimer in treated muscles. Representative bands are shown. Data represent mean±SD. * Statistically significant compared to control, p<0.05.

The *mdx* groups overexpressing dnMstat demonstrate increased body weight and wet weight of all muscles examined compared to age matched controls (**Fig. 4A, C, D**). The increases in limb skeletal muscle size were comparable to previously described approaches to myostatin inhibition [3], [13], [14]. In the *mdx* 4H group, the masses of the tibialis anterior, gastrocnemius and quadriceps muscle in treated animals were 38%, 34% and 46% larger than control, respectively (**Fig. 4C**). The increase in muscle mass observed in these muscles persisted to eleven months of age in the *mdx* 11H group. While the *mdx* 11L and *mdx* 11H muscle weights were not statistically different, both were greater than control muscle weights and there was a notable trend toward a larger magnitude of muscle growth when myostatin inhibition was initiated at one month of age (*mdx* 11H) in comparison to the neonatal period (*mdx* 11L) (**Fig. 4D**). The heart weight of treated *mdx* mice increased by 25% in the *mdx* 4H group and 22–42% in the *mdx* 11L and 11H groups (**Fig. 4C, D**). The heart/body weight ratio (mg/g) did not change in the *mdx* 4H group (4±0.1 vs 4±0.3). In the eleven month old groups, control *mdx* mice had a heart/body weight ratio of 4.72±0.1 which was not significantly different from the *mdx* 11H group (4.6±0.1). The *mdx* 11L group had a ratio of 4.2±0.1 which is less than controls. This is most likely because the increase in body weight in this group slightly outpaced the increase in heart weight.

Serum creatine kinase (CK) is a measure of the condition of whole body skeletal muscle. In *mdx* mice and humans afflicted by Duchenne muscular dystrophy the absence of dystrophin leads to sarcolemmal damage and increased membrane permeability, thereby elevating serum CK. Myostatin inhibition resulted in a significant decrease in serum CK levels in the *mdx* 4H group compared to age matched control *mdx* levels (**Fig. 4B**); however, serum CK levels in the *mdx* 11L and 11H groups did not differ from eleven month old control *mdx* levels.

**Figure 5:**
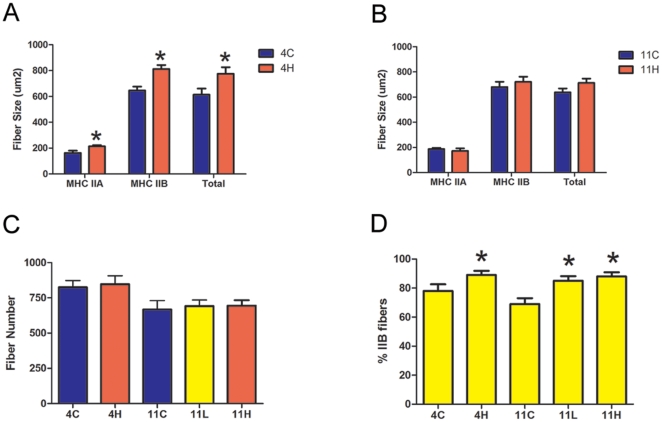
Morphological analysis of the *mdx* EDL. (a) In the *mdx* 4H group, the mean fiber size of myosin heavy chain (MHC) type IIA and IIB fibers increased which led to an overall increase in total fiber size. (b) At eleven months of age, the *mdx* 11H group did not demonstrate fiber size differences of either fiber type or total fibers compared to controls. (c) The number of fibers in the midbelly cross section of the EDL in all treated groups was not different from respective control fiber number. (d) There were 11% more type IIB fibers in the *mdx* 4H group while in the *mdx* 11L and 11H groups there were 16–19% more type IIB fibers. Data represent mean±SD. * Statistically significant compared to control, p<0.05.

**Figure 6:**
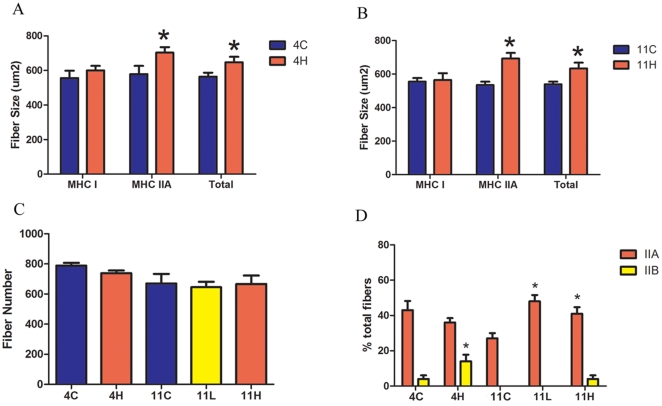
Morphological analysis of the *mdx* soleus. At the four month (a) and eleven month (b) timepoint myostatin inhibition did not change the mean fiber size of type I fibers but did increase the mean fiber size of myosin heavy chain (MHC) type IIA and total fibers. (c) Overexpression of dnMstat did not change the number of fibers in the midbelly cross section of the soleus. (d) In the *mdx* 4H group, the percentage of type IIB fibers was increased from 4% to 14%. At eleven months of age there is no significant difference in the percentage of IIB fibers among the experimental groups. However the *mdx* 11L and *mdx* 11H soleus contained 20–21% more type IIA fibers. Data represent mean±SD. * Statistically significant compared to control, p<0.05.

**Figure 7:**
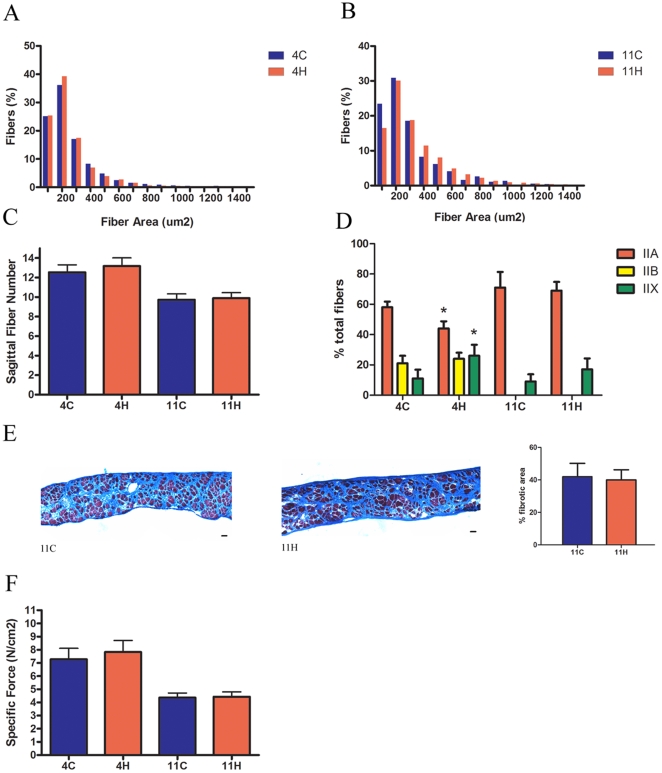
Morphological properties and force production of the *mdx* diaphragm. (a,b) Fiber size distributions were unaltered by myostatin inhibition in the *mdx* 4H and *mdx* 11H groups. (c) The number of fibers across the sagittal plane of the diaphragm, a measure of diaphragm fiber number, did not change at either time point. (d) In the *mdx* 4H diaphragm there were more type IIX fibers and less type IIA fibers. No changes in fiber type were observed in the *mdx* 11H group. (e) Masson's trichrome stained diaphragm sections did not demonstrate a difference in fibrotic area in the *mdx* 11H diaphragm in comparison to control (11C). (f) Overexpression of dnMstat did not improve specific force production in the diaphragm. Data represent mean±SD. * Statistically significant compared to control, p<0.05. Scale bar: 50 µm.

Immunohistochemistry was utilized to measure the cross sectional area (CSA) and to assess the myosin heavy chain (MHC) composition of muscle fibers from the EDL, soleus and diaphragm of the *mdx* groups. These skeletal muscles were chosen for analysis to determine if muscle groups with different fiber types and disease progression respond differentially to myostatin inhibition. The EDL represents a prototypical “fast” muscle type consisting of mostly fast glycolytic MHC type IIB fibers and the soleus represents a prototypical “slow” muscle type well populated with slow oxidative MHC type I fibers. The diaphragm contains a mixed fiber type distribution and is the mouse muscle in the *mdx* model most representative of human disease [24]. Both limb muscles exhibited an increase in muscle fiber size without a change in fiber number, with the exception of the EDL in the *mdx* 11H group, which did not hypertrophy (**Figs. 5** **,** **6**). Fiber sizes were further classified according to MHC type to determine which fiber type population accounted for the observed overall hypertrophy. In accord with prior studies that have found that myostatin preferentially acts on fast fiber types, the mean CSA of type IIA and IIB fibers was increased in the EDL while the mean CSA of only type IIA fibers was increased in the soleus (**Figs. 5A, B** **and** **6A, B**). The EDL had 11% more type IIB fibers in the *mdx* 4H group and 19% more type IIB fibers in the *mdx* 11H group (**Fig. 5D**). Similarly, the soleus had 13% more type IIB fibers in the *mdx* 4H group and 20% more type IIA fibers in the *mdx* 11H group (**Fig. 6D**). In both limb muscles there were not significant differences between the *mdx* 11L and *mdx* 11H groups. At both endpoints there were no differences in the diaphragm fiber size distribution or sagittal fiber number (**Figs. 7A, B, C**). The diaphragm exhibited an increase in type IIx fibers and decrease in type IIA fibers in the *mdx* 4H group while no difference in fiber type composition was found in the long term groups (**Fig. 7D**). The *mdx* 11H diaphragm was further analyzed by Masson's trichrome staining to assess the extent of connective tissue infiltration. As shown in Fig. 7E, the proportion of fibrotic area in the diaphragm was unchanged by long term overexpression of dnMstat.

The function of the EDL, soleus and diaphragm was assessed to determine if overexpression of dnMstat enhanced muscle strength. The *mdx* 4H EDL exhibited increased absolute tetanic force production comparable to the increased CSA resulting in no change in force normalized to CSA (specific force) (**Table 2**). In contrast, the *mdx* 4H soleus generated 55% greater tetanic force and 66% higher specific force, while force production in the diaphragm was unchanged (**Table 3** **,** **Fig. 7E**). These data indicate short term myostatin blockade improved force generation capacity of the limb muscles (soleus and EDL) but not the heavily utilized diaphragm. At eleven months of age, the soleus continued to demonstrate increased absolute and specific force generation and the effect was more pronounced in the *mdx* 11H group (**Table 3**). While CSA was increased in the *mdx* 11L EDL, tetanic force production was unchanged (**Table 2**). The absolute strength of the EDL was slightly increased in the *mdx* 11H group, yet the specific force declined by 21%. The *mdx* 11H diaphragm did not demonstrate any difference in force production (**Fig. 7E**). Thus in long term groups myostatin inhibition led to significantly improved force production only in the soleus muscle.

**Table 2:** Functional properties of EDL muscle in *mdx* groups.

	*Mdx* 4C	*Mdx* 4H	*Mdx* 11C	*Mdx* 11L	*Mdx* 11H
Mass (mg)	13.9±2.4^a^	19.8±2.2^b^	16.1±1	17.9±1.5^b^	22.7±1.4^b^
CSA (mm^2^)	2.2±0.3	2.9±0.5^b^	2.5±0.1	2.7±0.2^b^	3.5±.02^b^
Tetanus (mN)	360.4±33.9	489±30.4^b^	452.3±66	462.6±54	516.1±58.3^b^
Specific Force (N/cm^2^)	17.1±1.6	17.7±1.5	18.2±3.0	17.1±1.3	15.0±1.2^b^

a Data represent mean±SD from male four month old control *mdx* (n = 6), *mdx* 4H (n = 6), eleven month old control *mdx* (n = 6), *mdx* 11L (n = 6) and *mdx* 11H (n = 4).

b Statistically significant compared to control, p<0.05.

**Table 3:** Functional properties of soleus muscle in *mdx* groups.

	*Mdx* 4C	*Mdx* 4H	*Mdx* 11C	*Mdx* 11L	*Mdx* 11H
Mass (mg)	12.4±2.7^a^	16.4±2.5^b^	11.4±1.1	17.7±1.0^b^	18.2±2.1^b^
CSA (mm^2^)	2.1±0.7	1.8±0.2	2.1±0.2	3.1±0.3^b^	3±0.3^b^
Tetanus (mN)	160.6±35.4	248.6±32.4^b^	158.1±20.4	268.1±19.7^b^	325.2±33.0^bc^
Specific Force (N/cm^2^)	8.3±3.0	13.8±2.3^b^	7.7±1.5	8.7±1.2	10.9±2.4^b^

a Data represent mean±SD from male four month old control *mdx* (n = 6), *mdx* 4H (n = 6), eleven month old control *mdx* (n = 6), *mdx* 11L (n = 6) and *mdx* 11H (n = 4).

b Statistically significant compared to control, p<0.05.

c Statistically significant compared to *mdx* 11L, p<0.05.

To examine the interplay between myostatin and insulin like growth factor-I (IGF-I), quadriceps muscle homogenates from the *mdx* 4H group were analyzed using immunoblotting for myostatin and ELISA for IGF-I content. There was an approximately 60% increase in mature myostatin C-dimer in treated muscles (**Fig. 4E**) while there was no difference in IGF-I content (39 ng/g in control muscles vs. 34 ng/g in treated muscles). This finding is consistent with the report of IGF-I inducing myostatin expression but not vice versa [25].

**Figure 8:**
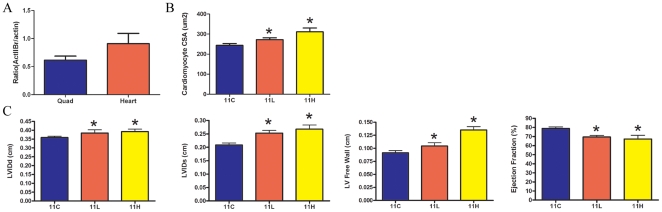
Cardiac properties in long term mdx mice. (a) Protein levels of the activin IIB receptor were similar in the *mdx* heart and quadriceps. (b) Increased mean cardiomyocyte cross sectional area (CSA) was noted in the *mdx* 11L and 11H groups. (c) Echocardiography demonstrated increased left ventricle internal diameter during diastole (LVIDd), increased left ventricle internal diameter during systole (LVIDs), increased left ventricle (LV) free wall diameter and decreased ejection fraction in treated animals. There was no difference between the *mdx* 11L and 11H groups across the reported parameters. Both groups were significantly different from control (11C). Data represent mean±SD. *Statistically significant compared to control, p<0.05.

The functional effect of myostatin inhibition on the dystrophic heart was determined by echocardiography. Eleven month old control *mdx* hearts and quadriceps were probed for levels of activin IIB receptor. The protein levels of activin IIB receptor in these tissues were similar (**Fig. 8A**). A myostatin inhibitor dose dependent increase of wet heart weight and mean cardiomyocyte CSA was observed in treated animals (**Figs. 4C, 4D** **,** **8B**). In the *mdx* 11H group ejection fraction was reduced by 11% and the left ventricular free wall was 43% thicker (**Fig. 8C**). Long term expression of dnMstat also increased LV diastolic and systolic internal diameter, which are features of the dilated cardiomyopathy inherent to the *mdx* mouse model that develops gradually over the lifespan of the animal [26], [27].

## Discussion

Persistent myostatin inhibition through AAV mediated expression of a novel myostatin inhibitor from the liver results in increased skeletal muscle mass in normal and *mdx* mice. It is important to note that not only does liver secretion result in body wide inhibition of myostatin, but it also allows for transgene stability for long periods of time which may not be possible with skeletal muscle delivery. Initiating myostatin inhibition in neonatal *mdx* animals resulted in a low level of circulating dnMstat while injection of one month old *mdx* animals resulted in a higher level (**Fig. 1E**). The dose of inhibitor is abbreviated as “L” for low dose and “H” for high dose which is preceded by the age of the animal at analysis. In an inhibitor dose dependent manner, skeletal muscle mass was increased in the *mdx* 11L and 11H groups. The skeletal muscle mass gains in our study are comparable to the administration of myostatin neutralizing antibody or propeptide, surpass prior studies of AAV mediated systemic myostatin inhibition and approach the muscle growth observed in *mdx*/myostatin knockout transgenic mice [3], [13], [14], [28].

Since evidence indicates myostatin acts on both satellite cells and adult fibers, increased muscle mass could occur via hypertrophy of existing myofibers, or via fomation of new fibers (hyperplasia). In previous studies, the deletion of myostatin by gene targeting promoted both hypertrophy and hyperplasia of skeletal muscle, whereas postnatal inhibition of myostatin induced hypertrophy only. The dual mechanism of increased muscle mass was also apparent in *mdx* mice crossed to myostatin null mice throughout life [7]. In the current study, in all muscles examined, muscle growth was due to hypertrophy rather than hyperplasia. This finding supports the notion that the prenatal absence of myostatin leads to both hypertrophy and hyperplasia in skeletal muscle, while postnatal inhibition of myostatin promotes muscle growth primarily by hypertrophy [29]–[31].

The absence of myostatin in knockout animals has been shown to skew the fiber type composition of the soleus and EDL toward faster MHC isoforms [32]. However when anti-myostatin antibodies were administered to two month old severe combined immunodeficient mice or myostatin was postnatally inactivated using a Cre-LoxP system in four month old mice there was no change in MHC profile of the soleus and EDL [29], [32]. Thus this is the first report of myostatin inhibition leading to an alteration in the MHC content of postnatal skeletal muscle. Reasons for the discrepancy between our findings in C57 Bl/6 animals and previous reports may be due to the efficacy of the inhibitor modality or the age of the animals when myostatin inhibition was initiated. Notably, there was a shift toward faster MHC isoforms in the soleus of the C57 neonates group but not in the C57 adults group. These results suggest there is a critical period of muscle development prior to three months of age during which myostatin can postnatally regulate fiber specification.

Fibertyping of 1 month old *mdx* soleus revealed 30% type I fibers and 70% type IIA fibers which is consistent with previous reports [33]–[35]. The absence of type IIB fibers at the initiation of myostatin inhibition at one month of age suggests a novel role for myostatin in the specification of fiber type. Rather than a selective survival advantage of fast fiber types, there appears to be an active switching toward fast MHC isoforms which is likely mediated by a mechanism acting on existing fibers as fiber number did not change. A potential mediator of this process is the presence of a large active satellite cell population. Rapid postnatal muscle growth in C57 Bl/6 animals is partially driven by the fusion of satellite cells to existing myofibers while in *mdx* animals there is a large scale degeneration-regeneration phase at 4–6 weeks of age followed by continual replacement of degenerated fibers throughout adult life. The observation of fiber type switching in neonatal C57 mice and all *mdx* animals suggests an activated homogenous satellite cell pool may contribute to the process of fiber specification influenced by myostatin. Another possibility is that there may be separate “slow” and “fast” satellite cell subpopulations and the selective activation of the “fast” subpopulation by myostatin inhibition may be populating the skeletal muscles with fast fiber types. Alternatively, the observed changes in MHC isoform expression may be due to direct action on muscle fibers and not involve satellite cells. Further experiments are necessary to delineate the mechanism and precise timing of fiber type switching of normal skeletal muscle induced by myostatin inhibition.

Immunoblotting of C57 Bl/6 muscle one week after viral injection revealed signaling changes consistent with myostatin inhibition. When the biologically active form of myostatin engages the activin IIB receptor an intracellular signaling cascade results in phosphorylation of Smad 2/3. Phosphorylated Smad 2/3 then translocates to the nucleus to suppress expression of myogenic factors and cell cycle progression in satellite cells. Other key regulators of muscle size have been implicated in the myostatin signaling pathway such as the serine/threonine kinase Akt and the MAP kinases ERK, JNK and p38 [25], [36]–[38]. In C57 Bl/6 mice overexpressing dnMstat there was a decrease in the levels of phosphorylated Smad 2/3 as was seen in C2C12 cells incubated with anti-myostatin antibody [3]. In our study there was also a decrease in JNK phosphorylation and an increase in Akt phosphorylation following acute inhibition in C57 Bl/6 animals. In agreement with our observations is a recent study of myotube hypertrophy which found increased levels of Akt phosphorylation following adenoviral overexpression of the myostatin propeptide [39]. These changes suggest muscle growth due to myostatin inhibition is partially mediated by the Smad 2/3, Akt and JNK signaling pathways and indicate that myostatin is functionally inhibited by dnMstat. Unaltered IGF-I content in treated muscles supports a mechanism of increased Akt phosphorylation achieved through the inhibition of myostatin rather than increased IGF-I. Further, the increase in activated myostatin in treated muscle indicates that under conditions of suppressed myostatin signaling there may be compensatory upregulation and/or increased local activation of myostatin to supercede the blockade. The upregulation of myostatin may be mediated through a Smad7 dependent auto-regulatory loop activated by the blockade of myostatin signaling [40], but this was not addressed in the current study.

In *mdx* animals myostatin inhibition was extremely effective at increasing muscle size and strength in the short term, however long term analysis yielded mixed results. Previous studies that have used antibodies directed against myostatin, propeptide injections or AAV mediated overexpression of an inhibitor have demonstrated increased muscle mass and absolute force production following 3–4 months of myostatin inhibition in young animals (1–3 months old) [3], [13], [14]. Our analysis of *mdx* animals at four months of age following three months of myostatin inhibition is consistent with previously published data. There was an increase in the mass of all skeletal muscles examined and both the soleus and EDL exhibited increased force production. However, following 10–11 months of dnMstat expression, myostatin inhibition did not result in functional benefit in the diaphragm or EDL. This implies myostatin inhibition as monotherapy will not be sufficient to attenuate dystrophic changes in the most severely affected skeletal muscles. In the *mdx* 11H group the treated diaphragm had equivalent force production and fibrotic replacement of muscle compared to controls (**Fig. 7**). As the diaphragm is more heavily utilized than limb muscles in mice it demonstrates early degenerative changes, progressive loss of contractile function and ten fold higher collagen content than other skeletal muscles [24]. Previous long term studies examining the *mdx* diaphragm in the context of reduced myostatin signaling have reported some histological benefit, but have not demonstrated functional improvement [7], [15]. While the *mdx*/Mstat KO diaphragm demonstrates improved histopathology at a later timepoint (18 months of age) than examined in this study, it is unclear if the apparent histological improvement correlates with enhanced function and if such changes are possible outside the context of knockout animals. Thus while long term myostatin inhibition improves mild dystrophy, as represented by the *mdx* limb muscles, it does not sufficiently drive muscle growth and reduce muscle fibroblast activity to compensate for severe dystrophy, as represented by the *mdx* diaphragm.

Functional analysis of the slow twitch soleus muscle and fast twitch EDL muscle revealed divergent effects of long term myostatin inhibition. The soleus muscle contains fiber types more representative of human skeletal muscle, and so the observed increase in specific force maintained in long term groups is an encouraging finding for clinical translation. This result may be attributable to a fiber type shift toward faster MHC isoforms. However, it is not clear if human skeletal muscle will shift in the same manner. At eleven months of age the *mdx* 11H EDL had decreased specific force production. These findings are analogous to the myostatin knockout mouse which demonstrates reduced specific force of the EDL but not the soleus [41], [42]. The exact etiology of reduced specific force output in the EDL is currently unknown. The documented abnormalities of the myostatin knockout EDL, such as a decrease in the number of mitochondria per fiber [41], are not sufficient to explain a drop in specific force production. Modulation of the TGF- β/Smad signaling pathway in myostatin knockout mice or long term inhibition in *mdx* mice results in reduced specific tension of the EDL that is of questionable therapeutic utility [41]. This effect appears to be confined to fast fiber types as the soleus had increased specific force in *mdx* groups. Further experiments are required to identify the mechanism of fiber type dependent impaired force generation due to myostatin blockade.

The lack of hypertrophy of type I fibers in response to myostatin inhibition in mice predicts that in humans a treatment that solely blocks myostatin regardless of modality will be much less effective than in preclinical mouse studies. In comparison to rodents human skeletal muscle has far fewer fast fibers and predominately consists of slow fibers. For example, the human soleus is entirely composed of type I fibers while mouse soleus consists of approximately 60% IIA fibers and 40% I fibers [43]. Importantly, human locomotor skeletal muscle does not contain type IIB fibers [44]. Type IIB fibers contain the highest density of activin IIB receptors, endogenous myostatin promoter activity and expression of myostatin in normal muscle [42], [45]. Therefore type IIB fibers are presumably the most responsive to myostatin mediated signaling. In this study we demonstrate that IIA fibers also undergo hypertrophy in response to myostatin inhibition in the soleus and EDL. This effect is critical for human therapy, as in concert with a fiber type shift to faster MHC isoforms hypertrophy of IIA fibers could lead to increased force production despite the near absence of fiber types most responsive to myostatin inhibition.

It is imperative to establish the safety of myostatin inhibition on the dystrophic heart because cardiomyopathy is nearly ubiquitous by 18 years of age in humans with Duchenne muscular dystrophy [46]. The absence of myostatin has been previously reported to have no effect on heart function in myostatin knockout animals or *mdx*/Mstat KO transgenics [47], [48].The studies addressing this issue have been either short term or relied on genetic crosses which have limited clinical relevance. Wagner et al. crossed *mdx* animals with myostatin knockout animals and did not observe any change in heart size or functional parameters at 24 months of age in *mdx*/Mstat KO animals [48]. However, the *mdx* mice in this study did not develop dilated cardiomyopathy at 24 months of age as *mdx* mice were indistinguishable from normal C57 Bl/6 controls across functional parameters. The absence of dilated cardiomyopathy in old *mdx* mice is in contrast to other studies in which echocardiographic changes suggestive of DCM have been first noted at 9–11 months of age and progress to a severe decline in ejection fraction to 35% at 21 months of age [26], [27], [49], [50]. In our study, myostatin inhibition did not affect heart weight in normal animals. However in treated *mdx* animals there was a 20% increase in heart weight at four months of age and a 40% increase in heart weight at eleven months of age. It is not likely that the increased heart weight is simply due to an increase in size in proportion to body weight. Barton et al. examined *mdx* mice that overexpressed the positive growth factor IGF-I only in skeletal muscle without systemic effects [12]. In this study an increase in skeletal muscle mass and body weight were observed without altering heart weight.

While the impact of myostatin inhibition on the progression of dilated cardiomyopathy in the *mdx* mouse or in more severe dystrophic cardiomyopathy is unknown, further investigation is necessary prior to implementing myostatin based therapies to treat disease conditions that include cardiac involvement. Differences in heart size or function in the *mdx* model following myostatin inhibition may not have been observed previously due to less effective myostatin inhibition or short term treatment regimens [3], [13], [14]. A possible mechanism for the observed cardiac hypertrophy is reduced myostatin signaling through the activin IIB receptor which is expressed at similar levels in the heart compared to skeletal muscle (**Fig. 8A**). Prior reports of the involvement of myostatin in cardiac remodeling include the upregulation of myostatin in cardiomyocytes bordering an infarcted region in sheep heart and in the left ventricle of volume overloaded rat heart [51], [52]. These data suggest that while myostatin is not involved in regulation of cardiomyocyte size in the normal heart it may be an important negative regulator of cardiomyocyte size under pathological conditions that trigger cardiac remodeling. Loss of myostatin signaling in these conditions may result in acceleration of cardiomyocyte hypertrophy and ultimately progression to heart failure. Further studies are needed to determine how the observed alterations in heart mass and function evolve over time.

Liver mediated overexpression of dnMstat has several important advantages over prior experimental approaches aimed at inhibiting myostatin. A clinical trial utilizing a neutralizing antibody directed at myostatin did not improve muscle function or increase muscle mass in adult patients with muscular dystrophy [53]. If implemented into clinical practice, any approach that involves injection of protein based inhibitors such as an antibody, myostatin propeptide or soluble activin IIB receptor would necessitate lifelong therapy while the use of AAV based inhibitors delivered to the liver requires only a single injection to achieve high levels of persistent transgene expression. There is limited possibility of immune recognition of the transgene product as dnMstat is identical to the endogenous propeptide with the exception of a single point mutation. In addition, there is evidence that liver production and secretion may induce tolerance to a foreign peptide [54]. The activin IIB receptor approach and other non-myostatin specific binding proteins such as follistatin may have limited clinical applicability since TGF-beta signaling in multiple organ systems may be impaired due to the broad tissue distribution of the activin IIB receptor [55].

Liver transduction also avoids the technical hurdle of direct transduction of dystrophic muscle and offers indefinite systemic delivery of therapeutic transgenes. In addition to the inherent difficulty of gene transfer to the entire musculature in large animal models and humans, long term transduction of dystrophic muscle by AAV without membrane stabilization is not feasible (unpublished observations by E.R. Barton and H.L. Sweeney, [20], [21]). This observation is contrary to a report describing myostatin inhibition following intramuscular injection of AAV 2/1 for over two years [15]. However there was no evidence presented that the transgenes were present in circulation or muscle at the study endpoint. Another possibility is that the liver was transduced in addition to short term limb muscle transduction as the promoter used in this study was not identified.

Our approach could be translated to larger animals by engineering species appropriate transgenes. The canine models of Duchenne muscular dystrophy more accurately recreate the clinical course of human disease than the *mdx* mouse and the liver has been successfully therapeutically transduced by AAV in a canine model of hemophilia B [56]–[59]. The dystrophic canine may be a more predictive indicator of the possible human efficacy of dnMstat than the *mdx* mouse. Even when the serum levels of the inhibitor were relatively low in the *mdx* 11L group, impressive gains in skeletal muscle mass and fiber type switching were observed. Myostatin inhibition via liver targeted gene transfer is highly adaptable to future gene therapy studies and has the potential to attenuate muscular dystrophy in large animal models.

## Methods

### Vector Production

The myostatin gene was isolated from mouse cDNA and the C terminal region from nucleotide 825–1131 of the reading frame was deleted via PCR. Splicing by overlap extension PCR was then used to introduce a D76A mutation that results in a peptide resistant to proteolytic activation. The resulting mutant transgene was named dominant negative myostatin (dnMstat) and was cloned into an AAV transfer vector with a liver specific promoter (α_1_-antitrypsin promoter with ApoE enhancer, kindly provided by Dr. Katherine High). The LSP has been used previously to express Factor IX from the liver of mice [57]. In addition, a control vector was cloned containing the LSP and green fluorescent protein (GFP). AAV pseudotype 2/8 was produced by the University of Pennsylvania Vector Core as previously described [22].

### Viral Injection of Mice

The virus used in all experiments was AAV2/8 LSP.dnMstat. The delivery method varied according to the age of the animal being treated. Neonatal mice were injected via a subxyphoid approach with 1E12 GC of virus following cryosedation as described previously [60]. To deliver virus to the liver of adult mice, intravenous tailvein injection was performed. Animals were sedated with a ketamine-xylazine mixture. Following sedation 1E12 genome copies of virus diluted in 300 ul saline was delivered into the tail vein. Non-viral controls were injected with 300 ul saline. Animals were sacrificed at the designated endpoints and the muscles frozen for histology, biochemical or functional analysis. All mouse experiments were approved by the University of Pennsylvania Animal Care and Use Committee.

### RT-PCR for Transgene Expression

The heart, diaphragm, liver, quadriceps, lung and kidney were dissected from mice two weeks post viral injection to assess the tissue specificity of the liver specific promoter. Tissues were crushed on a mortar and pestle cooled by dry ice and total RNA was isolated from muscle homogenates by RNA TRIZOL extraction (Gibco-BRL). Extracted RNA was treated with DNase (Ambion) to remove residual AAV genomes that may interfere with PCR analysis. 150 ng of RNA from each sample was subjected to single strand reverse transcription and the resulting cDNA was used in a PCR reaction with Taq polymerase (Applied Biosystems, Foster City, CA). The primers for dnMstat were forward 5′ AGG-CAC-TGG-TAT-TTG-GCA-GA 3′ and reverse 5′ GAA-CCT-GAA-ACA-TAA-AAT-GA 3′. The primers for beta-actin were forward 5′ ATC-ACT-ATT-GGC-AAC-GAG-CG 3′ and reverse 5′ ACT-CAT-CGT-ACT-CCT-GCT-TG 3′. The resulting PCR reactions were run on a 2% agarose gel with ethidium bromide and visualized under UV light.

### Muscle Morphology

For morphological analysis muscles were embedded in Optimal Cutting Temperature compound (Sakura Finetek, Torrance, CA) and frozen in liquid nitrogen cooled isopentane. Ten micron thick sections were cut and the resulting slides were stored at −20°C. Immunohistochemistry was employed to determine the fiber sizes, fiber number and myosin heavy chain (MHC) composition of examined muscles as described previously [12]. The myosin heavy chain (MHC) antibodies used to determine the MHC composition of selected muscles were type I (BA-F8) at 1∶50, type IIA (SC-71) at 1∶10 and type IIB (BF-F3) at 1∶3. Sections were blocked in 5%BSA/PBS and then incubated overnight in 5%BSA/PBS containing in a rabbit anti-laminin monoclonal antibody diluted 1∶100 (Neomarkers, Fremont, CA) and a MHC primary antibody at the dilutions described above. Following washes in PBS, sections were incubated in appropriate secondary antibodies (Invitrogen, Carlsbad, CA) for one hour in the dark at room temperature. Slides were washed and mounted with Vectashield with DAPI. All images were captured and processed on a Leitz DMRBE fluorescent microscope (Leica, Bannockburn, IL) equipped with a MicroMAX digital camera system (Princeton Instruments, Trenton, NJ). Open Lab imaging software (Improvision, Waltham, MA) was used for further analysis.

### Immunoblotting

For Western blotting muscles were dissected, weighed and snap frozen in liquid nitrogen. Muscles were crushed in a mortar and pestle on dry ice. Samples were then homogenized in lysis buffer [5 mM EDTA pH = 8, 50 mM Tris Cl pH 8,150 mM NaCl, 0.1% SDS, 1% Triton X-100, 0.5% deoxycholate, 50 mM dithiothreitol and Complete Protease Inhibitor Cocktail (Roche, Indianapolis, IN)]. Following incubation on ice for 10 min the protein homogenates were centrifuged at 12,000 RPM for 10 min. The supernatant was used for subsequent Western blotting. Protein concentration was determined by the Bio-Rad Protein Assay (Bio-Rad, Hercules, CA). 50 ug of protein or 1.5 µL of serum was mixed with 2x SDS loading buffer [130 mM Tris, pH = 8, 20% glycerol, 4.6% SDS, 2% DTT, 0.02% bromophenol blue], denatured at 95° for ten minutes and loaded onto 4–20% PAGEr Gold Precast Gels (Lonza, Rockland, ME). Proteins were transferred via the iBlot Dry Blotting System onto nitrocellulose membranes (Invitrogen, Carlsbad, CA). The membrane was then blocked with 5% non fat dry milk in Tris buffered saline containing 0.05% Tween 20. Immunoblotting was performed to detect myostatin N terminus (1∶1000, kindly provided by Dr. Se Jin Lee), phospho-Smad 2/3 (1∶500, Millipore, Temecula, CA), Smad 2/3 (1∶1000, Cell Signaling Technology, Danvers, MA), activin IIB receptor (1∶500, Sigma, St. Louis, MO), phospho-Akt and total Akt (1∶1000, Cell Signaling Technology, Danvers, MA), phospho-JNK and total JNK (1∶1000, Cell Signaling Technology, Danvers, MA), and actin (1∶2000, Sigma, St. Louis, MO). Appropriate horseradish peroxidase conjugated secondary antibodies were used at twice the dilution of the primary antibody (GE Healthcare, UK). Protein detection was performed by exposure to Super Signal West Pico Chemiluminescent Substrate kit (Pierce, Rockford, IL) and the band intensity was processed using densitometry software (Alpha Innotech, San Leandro, CA).

### Muscle Functional Analysis

The contractile properties of the soleus, extensor digitorum longus (EDL) and diaphragm muscle were measured. Mice were anesthetized with ketamine/xylazine (80 and 10 mg/kg body wt, respectively) and exsanguinated. Blood samples were allowed to clot, centrifuged at 2000×g for 20 minutes, and then stored at −80°C for creatine kinase measurement and immunoblotting. The EDL, soleus, diaphragm, and gastrocnemius muscles were removed, placed in a bath of Ringers solution gas-equilibrated with 95% O_2_/5% CO_2_. and were subjected to isolated mechanical measurements using a previously described apparatus (Aurora Scientific, Ontario, Canada) [61]. After determining optimum length (Lo) by supramaximal twitch stimulation, maximum isometric tetanus was measured. Upon completion of these measurements, muscles were weighed and rapidly frozen in melting isopentane for morphological analysis.

### *In Vivo* Transthoracic Echocardiography

M-mode echocardiography was performed on *mdx* animals at eleven months of age under ketamine/xylazine anesthesia using a 15-MHz phased-array probe connected to a Sonos 7500 echocardiographic machine (Philips Medical Imaging, Andover, Massachusetts). In brief, an M-mode cursor was positioned in the parasternal short-axis view perpendicular to the interventricular septum and posterior wall of the LV at the level of the papillary muscles, and M-mode images were obtained for measurement of LV end-diastolic and end-systolic dimension (LVDd and LVDs). The percentage of fractional shortening (%FS) was calculated from the equation %FS  =  [(LVDd–LVDs)/LVDd] x 100. The end diastolic and end systolic volumes, ejection fraction, cardiac output and stroke volume were calculated using the Teicholtz formulas [62]. The same sonographer (MS) was blinded to the treatment groups of the mice, performed all studies and the resulting calculations.

### Statistical Analysis

Comparison of means from each experimental group was accomplished by two tailed unpaired Student's t-test or one-way ANOVA with Student-Newman-Keuls *post hoc* analysis as appropriate. Numerical data is reported as mean ± standard deviation. P values <0.05 were accepted as significant.
